# Integrin-Linked Kinase Suppression Reduces SPARC's Effect on Intraocular Pressure and Extracellular Matrix

**DOI:** 10.1167/iovs.67.10.53

**Published:** 2026-08-21

**Authors:** Charles W. Guo, Seth Birrell, Sophie Baillargeon, Karl Koster, Bridger Jeppesen, Yuxi Zheng, Alexandra Castillejos, Archana Murali, Min Hyung Kang, Jonah Scott-McKean, Hyunpil Lee, Shigemi Matsuyama, Douglas J. Rhee

**Affiliations:** 1 Department of Ophthalmology and Visual Sciences, Case Western Reserve University, School of Medicine, Cleveland, Ohio, United States; 2 Department of Ophthalmology, University Hospitals Cleveland Medical Center, Cleveland, Ohio, United States; 3 Case Comprehensive Cancer Center, Cleveland, Ohio, United States

**Keywords:** glaucoma, extracellular matrix (ECM), secreted protein, acidic and rich in cysteine (SPARC), integrin-linked kinase (ILK), intraocular pPressure (IOP)

## Abstract

**Purpose:**

The molecular pathway for increased outflow resistance in the trabecular meshwork (TM) remains unknown. Secreted protein, acidic and rich in cysteine (SPARC) has been shown to regulate intraocular pressure (IOP) correlating to extracellular matrix (ECM) alterations. In other tissues, SPARC binds integrin-linked kinase (ILK) and regulates ECM organization through signaling cascades. We hypothesized SPARC regulates ECM proteins partly through ILK in the TM.

**Methods:**

Adenovirus carrying cDNA of human SPARC (Ad.SPARC) was used to overexpress SPARC and a lentivirus carrying short-hairpin RNA (shRNA) targeting human ILK (shILK) was used to inhibit ILK in live mice, primary human TM cells, and perfused human cadaveric anterior segments. IOP was measured in mice and human anterior segments. Selected ECM proteins were analyzed by immunoblotting and immunostaining.

**Results:**

SPARC overexpression elevated IOP 1.87 ± 0.50 millimeters of mercury (mm Hg; *P* = 0.009, *n* = 15) in mice. Coinfection with ILK inhibition reduced IOP by 3.64 ± 0.57 mm Hg (*P* < 0.001, *n* = 15) and histologically reduced ECM proteins compared to SPARC overexpression. In human anterior segments, SPARC overexpression elevated IOP 2.10 ± 0.25-fold (*P* = 0.021, *n* = 4), whereas addition of shILK attenuated SPARC's effect and decreased IOP 0.61 ± 0.31-fold (*P* = 0.015, *n* = 4). In human TM cells, Ad.SPARC infection elevated levels of SPARC and laminin. Coinfection with shILK reduced levels of ILK, collagen I, collagen VI, and laminin.

**Conclusions:**

ILK inhibition attenuated the effects of SPARC overexpression on IOP and ECM proteins, suggesting ILK signaling may contribute to the pathway of SPARC-mediated regulation of ECM homeostasis in TM. Future studies evaluating the downstream transcription factors may provide an elucidated pathway for POAG pathogenesis.

Primary open-angle glaucoma (POAG) is characterized by increased resistance to aqueous humor outflow within the trabecular meshwork (TM), resulting in elevated intraocular pressure (IOP) and optic nerve damage.^1^^–^^3^ In older patients, which is the majority of patients with POAG, the conventional pathway is responsible for >80% of aqueous outflow.^4^^,^^5^ As humans age, there is progressive accumulation of extracellular matrix (ECM) proteins within the juxtacanalicular region (JCT), increasing the resistance and reducing aqueous humor outflow.^2^^,^^6^

TGF-β is one of the mediators of ECM deposition and is significantly elevated in the aqueous humor of patients with POAG.^7^ TGF-β upregulates collagens and other fibrotic matrix components believed to be responsible for ocular hypertension.^7^^,^^8^ Accordingly, secreted protein, acidic and rich in cysteine (SPARC) is another ECM regulator protein highly expressed in the TM and downstream of TGF-β.^9^^,^^10^ SPARC-null mice demonstrate increased aqueous outflow with lower baseline IOPs, blunting TGF-β–induced ocular hypertension and implicating it as a key regulatory step.^11^^–^^14^ SPARC overexpression also increases IOP and ECM proteins and, conversely, SPARC suppression has the opposite effects.^14^^–^^16^ Although the correlational effect of SPARC on ECM components has been demonstrated, the intermediate molecular steps from receptor engagement to transcription factor activation remain to be elucidated.

In murine pulmonary fibroblasts, myoblasts, and epithelial lens cells, SPARC partly regulated the ECM through the activity of integrin-linked kinase (ILK).^17^^–^^19^ ILK is a serine/threonine kinase bound to the intracellular domain adjacent to the plasma membrane, and is responsible for the intracellular signaling controlling cytoskeletal organization and ECM assembly.^20^^,^^21^ In other models, ILK suppression inhibits AKT phosphorylation, downregulating collagen secretion and increasing matrix metalloproteinase (MMP) activity.^22^^,^^23^ The structural domains and parvin-binding interfaces of ILK are highly evolutionarily conserved, with humans and rats sharing 99.6% amino acid sequencing.^24^^,^^25^ We hypothesized SPARC may be exerting its effects in the TM through its interaction with ILK. In this hypothesis-generating study, we overexpressed SPARC and inhibited ILK to determine the effects on IOP, ECM proteins, and the actin cytoskeleton.

## Methods

### Animal Husbandry

All experiments were done in accordance with the ARVO Statement for the Use of Animals in Ophthalmic and Vision Research and received local Institutional Animal Care and Use Committee (IACUC) approval. C57BL/6 × 129/SvJ wild-type (WT) mice were originally obtained from E. Helene Sage of the Benaroya Research Institute at Virginia Mason (Seattle, WA, USA).^26^ Animals used in these experiments were both male and female animals between 6 and 8 weeks old and housed in the Animal Research Center of Case Western Reserve University. The baseline IOP in our WT mice is higher than other typical strains and aligns with other reported IOPs of 19.9 ± 2.9 millimeters of mercury (mm Hg).^11^

### Adenoviral and Lentiviral Constructs and Production

Adenoviral (Ad) vectors containing human SPARC gene (Ad.SPARC) were produced using the transpose-Ad adenoviral (Ad5) vector system (Qbiogene, Carlsbad, CA, USA) exactly as described in detail previously.^15^ A 973 bp human SPARC cDNA fragment was amplified by RT-PCR using human-specific primers, cloned into the pCR259 vector (Qbiogene) at Nhe1 and Xba1 restriction sites,^27^ and orientation was confirmed by PCR. The resulting Ad.CMV.h-SPARC plasmid, along with an empty vector control, were transformed in *Escherichia coli* (*E. coli*) DH5, amplified, and purified using the EndoFree Plasmid Maxi Kit (Qiagen, Valencia, CA, USA). Both constructs were transfected into HEK293 cells using the CalPhos Mammalian Transfection Kit (Clontech, Mountain View, CA, USA) and incubated at 37°C and 5% CO_2_ until cytopathic effect was observed. Viral supernatant was collected, and Ad5.hSPARC was purified and titered using the ViraBind purification and QuickTiter immunoassay kits (Cell Biolabs, San Diego, CA, USA). Both constructs contained the CMV promoter and replication-deficient adenoviral genes.

Lentiviruses carrying short-hairpin RNA (rhRNA) targeting human ILK (shILK) or control RNA (shCtrl) were constructed using a lentiviral shRNA kit (OpenBiosystems pLKO.1 vector system, Huntsville, AL, USA). Before transfection, HEK293T cells were plated at a density of 5.5 × 10^6^ cells in 100-mm plates. The day of transfection, DNA/Arrest-In complexes were developed by combining 9 µg of the DNA construct, 28.5 µg of packaging mix, and 187.5 µg of Arrest-In transfection reagent, in 2 mL of serum-free media. The mixture was incubated at room temperature (RT) for 20 minutes, and 3 mL of serum-free medium was added into the complexes. It was then applied to the cells (HEK293T), which were incubated at 37°C in a 5% CO_2_ chamber for 6 hours. After 6 hours, the transfection mixture was replaced with 12 mL DMEM (10% fetal bovine serum, 1× penicillin/streptomycin, and 1× L-glutamate). The cells were incubated under the same conditions for another 48 hours, after which the lentivirus-containing media were collected. The lentivirus was purified and quantified using the ViraBind Lentivirus Concentration and Purification Kit and QuickTiter Lentivirus Quantification Kit (Cell Biolabs Inc., San Diego, CA, USA). Verification of successful ILK knockdown was conducted in human TM cells with independent shRNA lentiviral particles purchased through Origene (TL320386V).

### Murine Intravitreal Injection

Mice were anesthetized with 5 uL/g anesthesia (Ketamine/Xylazine 20 mg/1.75 mg/mL; Phoenix Pharmaceutica, St. Joseph, MO, USA) and were positioned on an adjustable mount (Mouse and Neonatal Rat Adaptor; Stoelting Co., Wood Dale, IL, USA). The eye received one drop of 0.5% proparacaine and lubricating eye drops. Injections were done using a 35-gauge needle (NF35BL-2, World Precision Instruments, Sarasota, FL, USA) and a 10-uL micro syringe (Nanofil; World Precision Instruments), as described in detail previously.^13^ One treatment group was injected with Ad.SPARC + shCtrl to induce SPARC overexpression with a lentivirus control. The second treatment group was injected with Ad.SPARC + shILK to induce SPARC overexpression with ILK suppression. A vehicle control group containing Ad.Ctrl + shCtrl was also injected as a negative control group. A final titer of 6 × 10^6^ plaque-forming units (1.5 µL of 4 × 10^9^ pfu/mL) was achieved. The contralateral eyes were not injected and served as the untreated control.

### Murine IOP Measurement

IOP was measured by rebound tonometry as previously described.^14^ Measurements were conducted between 11:00 AM and 3:00 PM to control circadian effects on IOP.^28^ Mice were anesthetized and a TonoLab tonometer probe-tip was positioned 2 to 3 mm directly anterior and perpendicular to the central cornea (TonoLab; Colonial Medical Supply, Franconia, NH, USA), a method which has been previously demonstrated to be accurate.^11^ Measurements were taken between 4 and 7 minutes following the anesthetic injection.^29^^,^^30^ The IOP of both eyes were measured the day before injection, the day of injection, as well as days 3, 5, 7, 10, 13, and 15 after injection. The IOPs obtained the day prior, and the day of the injection were averaged and used as baseline IOP.

### Immunostaining

Mice were euthanized and immediately enucleated. Eyes were fixed in 10% formalin for 48 hours at RT, followed by 70% ethanol at 4°C. Eyes were then processed and embedded in paraffin in 6-µm thick sections. Removal from paraffin involved 3 washes in xylenes and sequential hydration in 100%, 95%, and 70% ethanol. Slides were then blocked with 1% normal goat serum (NGS) in 1× PBST (1× PBS, 0.1% Tween-20) for 45 minutes at RT. Primary antibody solutions were diluted in 1% NGS in 1× PBST (e.g., 1:100 ratio, 1 µL primary antibody per 99 µL NGS-PBST mix), and concentrations used are in the Table. Primary antibody was applied to sections overnight at 4°C. After incubation, sections were rinsed with 1% NGS in 1× PBST and then washed twice. Secondary antibodies were applied to sections for 2 hours at RT in the dark. After another wash, one drop of DAPI nuclear stain with Fluoromount was added. Confocal microscopy was performed within 24 to 48 hours of secondary antibody staining using an Olympus microscope and FluoView software. A 40× oil immersion objective lens was utilized to collect images of the mouse trabecular meshwork, and a 20× objective was used for human anterior segments. The intensity of fluorescence for all stains were kept uniform and unaltered across each protein target. ECM fluorescence was quantified by using ImageJ software, with five repeated measures within different areas of the TM. Fluorescence values were then normalized to a negative control treated with only secondary antibodies and DAPI. Comparisons between treatment groups were conducted after normalization.

**Table.** Primary and Secondary Antibodies Used in Immunoblot and Immunofluorescence

Primary Antibody	Company	Host Species	Dilution	Secondary Antibody	Company	Dilution
Immunoblot						
Collagen I	Rockland	Rabbit	1:1000	HRP antirabbit IgG	Promega	1:5000
Collagen VI	Santa Cruz Biotech	Rabbit	1:1000	HRP antirabbit IgG	Promega	1:5000
Fibronectin	Sigma-Aldrich	Mouse	1:1000	HRP antimouse IgG	Promega	1:5000
Laminin	Novus	Rabbit	1:1000	HRP antirabbit IgG	Promega	1:5000
B-actin	Invitrogen	Mouse	1:10,000	HRP antimouse IgG	Promega	1:5000
SPARC	Hematologic Tech	Mouse	1:10,000	HRP antimouse IgG	Promega	1:5000
ILK	Abcam	Rabbit	1:3000	HRP antirabbit IgG	Promega	1:5000
Immunofluorescence						
SPARC	Hematologic Tech	Mouse	1:500	Donkey antimouse 594	Molecular Probes	1:200
ILK	EMD Millipore	Rabbit	1:100	Donkey antirabbit 488	Molecular Probes	1:200
Collagen I	Rockland	Rabbit	1:100	Donkey antirabbit 488	Molecular Probes	1:200
Collagen IV	Rockland	Rabbit	1:100	Donkey antirabbit 488	Molecular Probes	1:200
Collagen VI	Rockland	Rabbit	1:100	Donkey antirabbit 488	Molecular Probes	1:200
Fibronectin	Sigma-Aldrich	Mouse	1:100	Donkey antirabbit 594	Molecular Probes	1:200
Laminin	Sigma-Aldrich	Mouse	1:100	Donkey antirabbit 594	Molecular Probes	1:200

### Human Perfused Anterior Segment

Human donor eyes (aged 42, 46, 55, and 57 years) with no known ocular pathology were processed within 24 hours postmortem and acquired from regional eye banks and perfused using the methods outlined by Oh et al.^15^ Human anterior segments were isolated by removing the iris, lens, and vitreous and mounted into a plexiglass chamber. The anterior segments were perfused at a constant flow rate of 2.5 µL/min with DMEM (Thermo Fischer-Gibco) containing 1% FBS, 1% L-glutamine (2 mM) penicillin (100 U/mL), streptomycin (100 U/mL), gentamicin (0.17 mg/mL), and amphotericin-B (0.25 µg/mL) under 5% CO_2_ at 37°C. The IOP of the perfused eyes were measured every 5 seconds using a pressure transducer (BD Medical, Franklin Lakes, NJ, USA), averaged every hour, and recorded using an automated computer system (National Instruments, Austin, TX, USA). After 24 hours of stable pressure, both eyes were injected intracamerally with Ad.SPARC composing of 100 µL containing 1 × 10^9^ infectious units per mL (ifu/mL).^31^ Forty-eight hours after the initial injection, a second injection of 100 µL containing 1 × 10^9^ ifu/mL of either shILK or shCtrl was administered. Eyes were selected randomly. After 2 weeks, the anterior segments were fixed and processed in paraffin for immunostaining.

### Human TM Cell Culture and Infection

Primary human TM cell cultures were derived from 11 separate donors (variable age range from 40–73, both male and female donors, and all without ocular medical history). The TM was dissected from the corneoscleral rims that were discarded after corneal surgery and segmented for use as primary cell lines in compliance with the tenets of the Declaration of Helsinki. The maintenance growth medium consisted of DMEM supplemented with 20% fetal bovine serum (FBS; Invitrogen-Gibco, Grand Island, NY, USA), 1% L-glutamine (2 mM), and/or 0.1% gentamicin (50 or 10 ug/mL). For each experiment, TM cultures from passage 4 or 5 were seeded into nine 6-well plates (Costar, Corning, NY, USA), allowed to grow to confluence at 37°C in a 5% CO_2_ atmosphere, and given an additional 2 to 3 days for post-confluence maturation in complete media as per our previously published protocol and in accordance with best published practices.^15^^,^^32^

For viral infection, TM cells were incubated in 2% FBS medium with 50 MOI of Ad.Ctrl or Ad.SPARC and 10 MOI of shCtrl or shILK for 18 hours as previously published.^15^ Subsequently, cultures were switched to 10% FBS medium for 24 hours. Cultures were then switched to serum-free medium for 24 hours, replaced with adenovirus-containing medium for a final 24 hours, after which the serum-free conditioned medium was collected for analysis.

### Immunoblot Analysis

Cultured TM cells were lysed, and protein concentrations were established using Bradford Protein Assay (Bio-Rad, Hercules, CA, USA). Equal quantities of proteins were mixed with 4× Reducing Buffer (250 mM of Tris base, 8% SDS, 40% glycerol, 0.002% bromophenol blue, and pH adjusted to 6.8) and incubated at 95°C for 5 minutes. SDS-PAGE was performed and loaded onto a gradient gel (Invitrogen NuPAGE 4–12% Bis-Tris). The gels were transferred into a nitrocellulose membrane with 0.45 µm pore size (Invitrogen) using a transfer device (Invitrogen, iBlot 2). The membrane was incubated in blocking buffer (1× TBST, 5% bovine serum albumin [BSA], 3% milk, and 2% NGS) for 1 hour, and then with a primary antibody (see the Table) overnight at 4°C. After approximately 12 hours of incubation, the membrane was washed and treated with horseradish peroxidase (HRP) secondary antibodies for 1 hour at RT. After incubation, the membrane was again washed and covered in 6 mL of SuperSignalWest Pico PLUS (Thermo Scientific) for 5 minutes. Membranes were imaged using the KwikQuant Digital Western Blot Detection System and protein density was quantified using ImageJ software (National Institutes of Health, Bethesda, MD, USA).

### Immunofluorescence Staining for f-actin

Primary TM cells cultured in 8-well slides were fixed using 4% paraformaldehyde in PBS (pH 7.4) at 4°C for 30 minutes and then washed in 1× PBS for 10 minutes twice at RT. The cells were permeabilized using 0.2% Triton X-100 in 1× PBS for 5 minutes, washed again in 1× PBS 2 times for 10 minutes, then blocked in 3% BSA in PBS for 1 hour at RT. Primary antibody was applied and incubated at 4°C overnight. After approximately 12 hours, the wells were washed 3 times with 1× PBS for 10 minutes. Secondary antibodies specific to the primary antibodies were applied in a 1:100 ratio, and nuclei were stained using DAPI (Invitrogen). F-actin was stained with Alexa Fluor 568 phalloidin (1:40; Invitrogen). Cells were analyzed and imaged using the Leica TCS SP2 spectral confocal laser scanning microscope (Leica Microsystems, Exton, PA, USA). After obtaining multiple fluorescent images, the structural change of f-actin in human TM cells was quantified using FibrilTool, which was developed to analyze the anisotropy and orientation of fibrillar structure in the cells and is a “plug-in” to Image J software.^33^ Five circular areas of interest measuring 20 × 20 pixels were measured within different areas of an image. Nuclear and membranous regions were avoided. Oversaturated and blurry images such that cellular filaments were indistinguishable were excluded. Anisotropic values were measured and reported by the FibrilTool according to published protocol.^33^

### Statistics

Statistical analyses were conducted using SigmaStat software version 1.0 (Jandel Corporation, San Rafael, CA, USA) or directly in Prism software version 11.0.2 (GraphPad, Boston, MA, USA). One-way analysis of variance (ANOVA) with post hoc Tukey analyses were used to assess for significant differences between multiple comparison groups. A paired *t*-test was used to calculate *P* values within one group at multiple time points. A *P* < 0.05 was considered statistically significant when testing two groups; (*) represents *P* < 0.05, (**) represents *P* < 0.01, (***) represents *P* < 0.001, and (****) represents *P* < 0.0001. All data were presented as the mean ± standard error.

## Results

### Effect of SPARC Overexpression and ILK Inhibition in Mice

**Figure 1.**
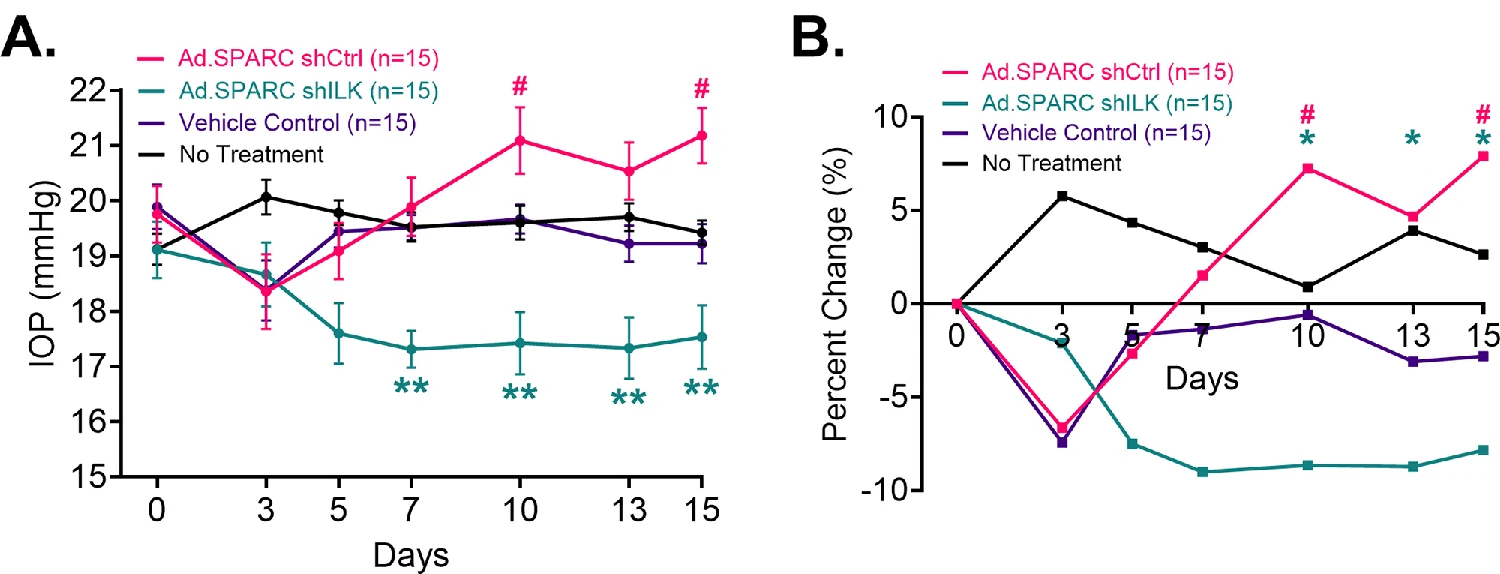
(**A**) IOP measurements of mice injected with Ad.SPARC + shCtrl (*n* = 15), Ad.SPARC + shILK (*n* = 15), or vehicle control (Ad.Ctrl + shCtrl, *n* = 15). The uninjected contralateral eyes served as control (“No Treatment”). # represents *P* < 0.05 comparing Ad.SPARC + shCtrl and non-treated eyes. * Represents *P* < 0.05 comparing Ad.SPARC + shILK to non-treated eyes. ** Represents *P* < 0.01. (**B**) Percent change differences compared to the baseline IOP.

**Figure 2.**
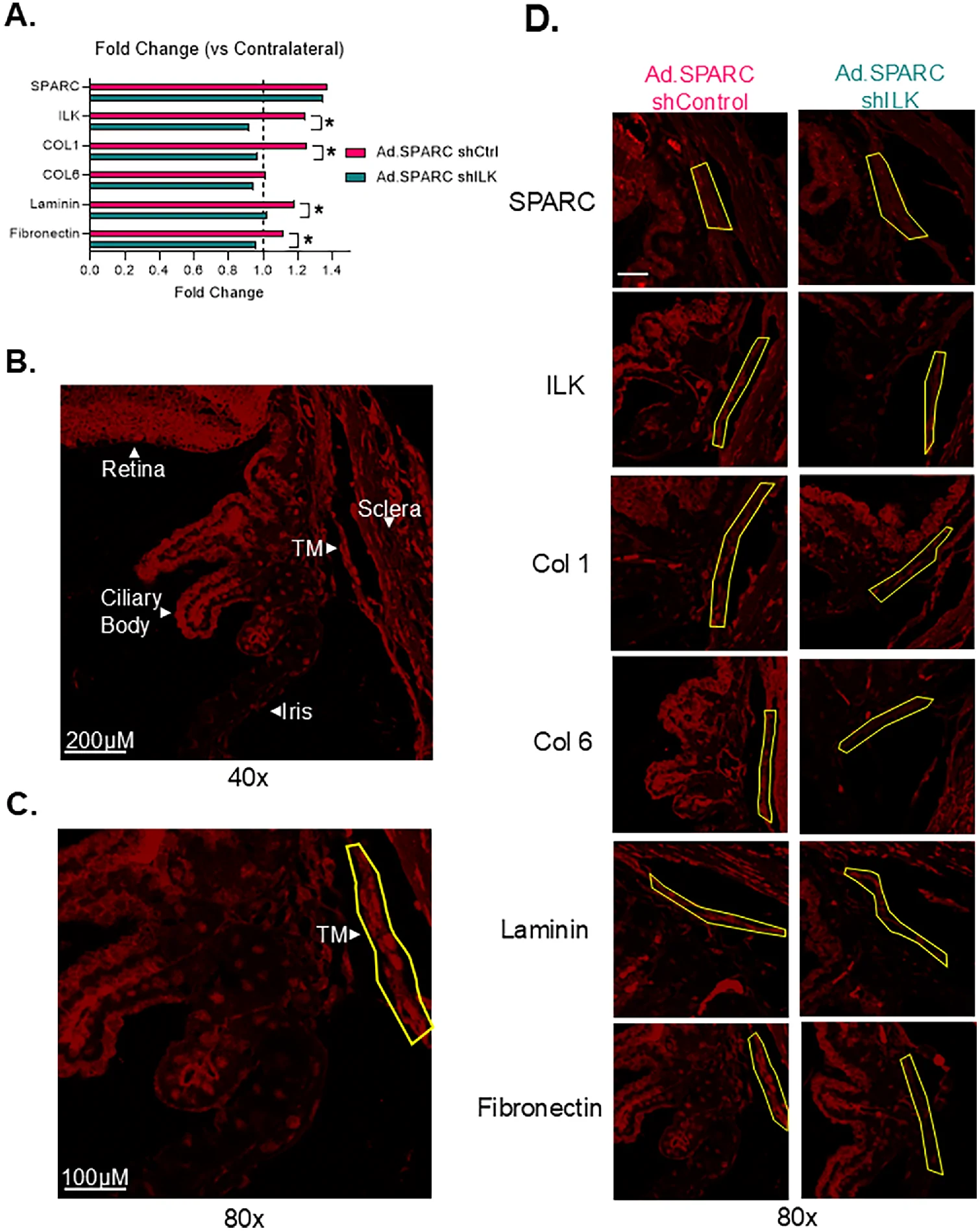
Immunostaining of ECM proteins in histologic sections of mouse eyes following injection. (**A**) Fluorescence intensity measurements were compared relative to the non-treated eye. (**B**) The 40× objective lens image with labeled structures. (**C**) The 2× digital zoom applied, with the *yellow bar* highlighting the TM region of interest. (**D**) Representative images with the TM highlighted. Brightness was adjusted uniformly across treatment groups for visibility. Quantification was performed on raw images. Supplementary Figure S1 shows additional DAPI and negative control images.

Fifteen days after viral infections, SPARC overexpression elevated IOP 1.75 ± 0.50 mm Hg (*P* = 0.005, *n* = 15) compared to the uninjected contralateral eyes (Fig. 1A). Comparatively, addition of ILK inhibition by shILK reduced IOP by 3.64 ± 0.57 mm Hg (*P* < 0.001, *n* = 15). The mice injected with shILK saw consistent IOP reductions below basal levels after 5 days, despite the elevated ocular pressure induced by SPARC overexpression (Fig. 1B). There were no significant differences between the vehicle controls and non-treated eyes, besides the initial IOP drop from the eye injection. Immunostaining of SPARC overexpressed eyes demonstrated elevated expression of ILK 1.25 ± 0.07-fold (*P* = 0.01, *n* = 8), Col I 1.26 ± 0.10-fold (*P* = 0.04, *n* = 8), and laminin 1.18 ± 0.06-fold (*P* = 0.01, *n* = 8) when compared with untreated controls in the juxtacanalicular region (Fig. 2A). Addition of shILK reduced levels of ILK 0.33 ± 0.02-fold (*P* < 0.01, *n* = 8), Col I 0.29 ± 0.03-fold (*P* = 0.03, *n* = 8), laminin 0.15 ± 0.05-fold (*P* = 0.02, *n* = 8), and fibronectin 0.16 ± 0.06-fold (*P* = 0.05, *n* = 7) despite the elevated levels seen in SPARC overexpression alone (see Fig. 2A). Representative TM histology was labeled and compared in Figures 2B to 2D. DAPI stains and negative control stains can be seen in Supplementary Figure S1, and co-staining of SPARC and ILK in Supplementary Figure S3. No qualitative changes were noted in cellularity or morphology. The overexpression of SPARC resulted in elevated IOP and ECM proteins in mice, whereas the subsequent suppression of ILK reduced IOP and ECM proteins below the untreated eyes.

### Effect of SPARC Overexpression and ILK Inhibition in Perfused Cadaveric Human Anterior Segments

**Figure 3.**
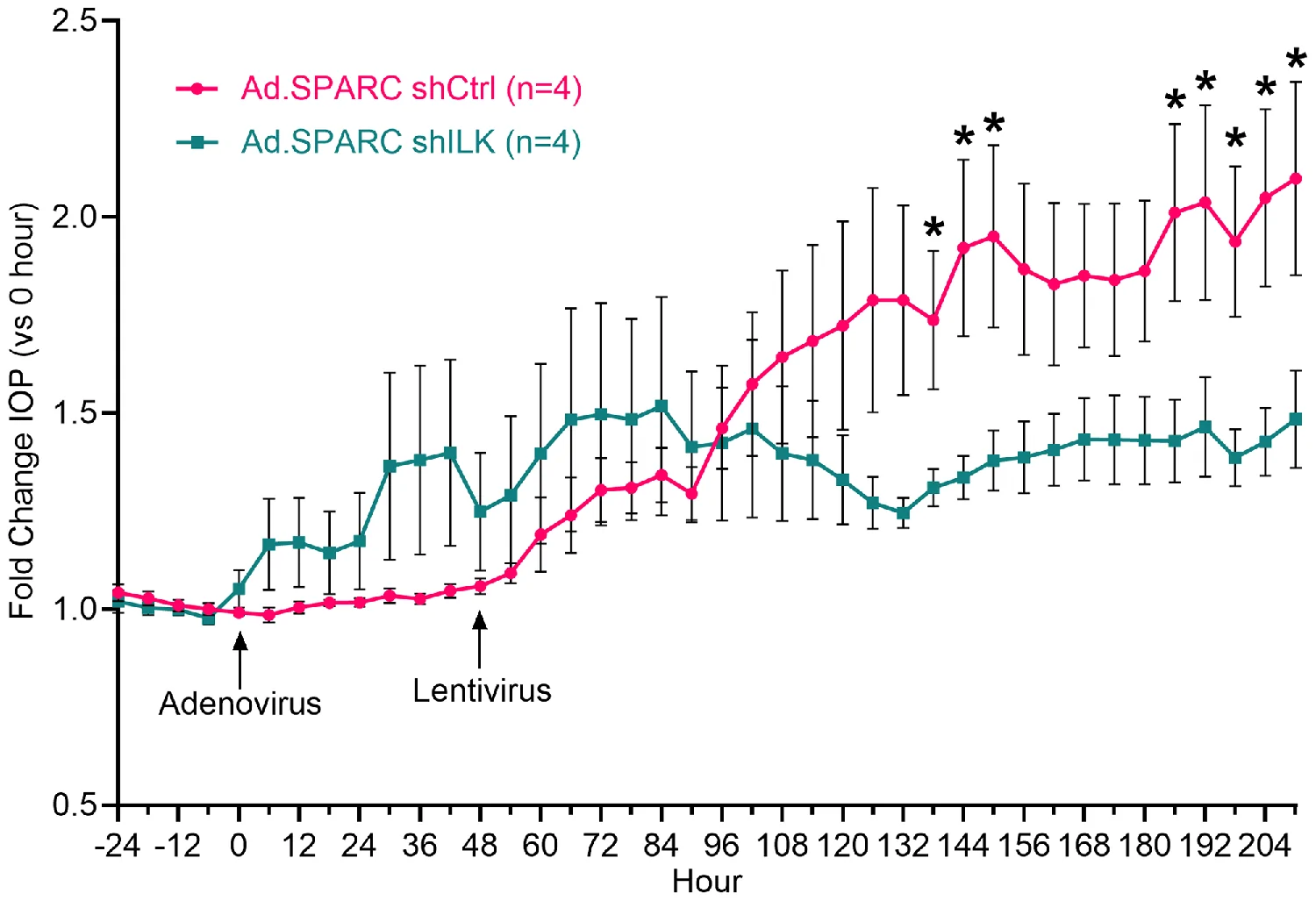
Effect of SPARC overexpression and ILK inhibition on IOP in perfused cadaveric human anterior segments (*n* = 4). Eyes reached stable pressure for 24 hours before intracameral injection of Ad.SPARC. After 48 hours, shCtrl (Control) or shILK (anti-ILK) was injected.

**Figure 4.**
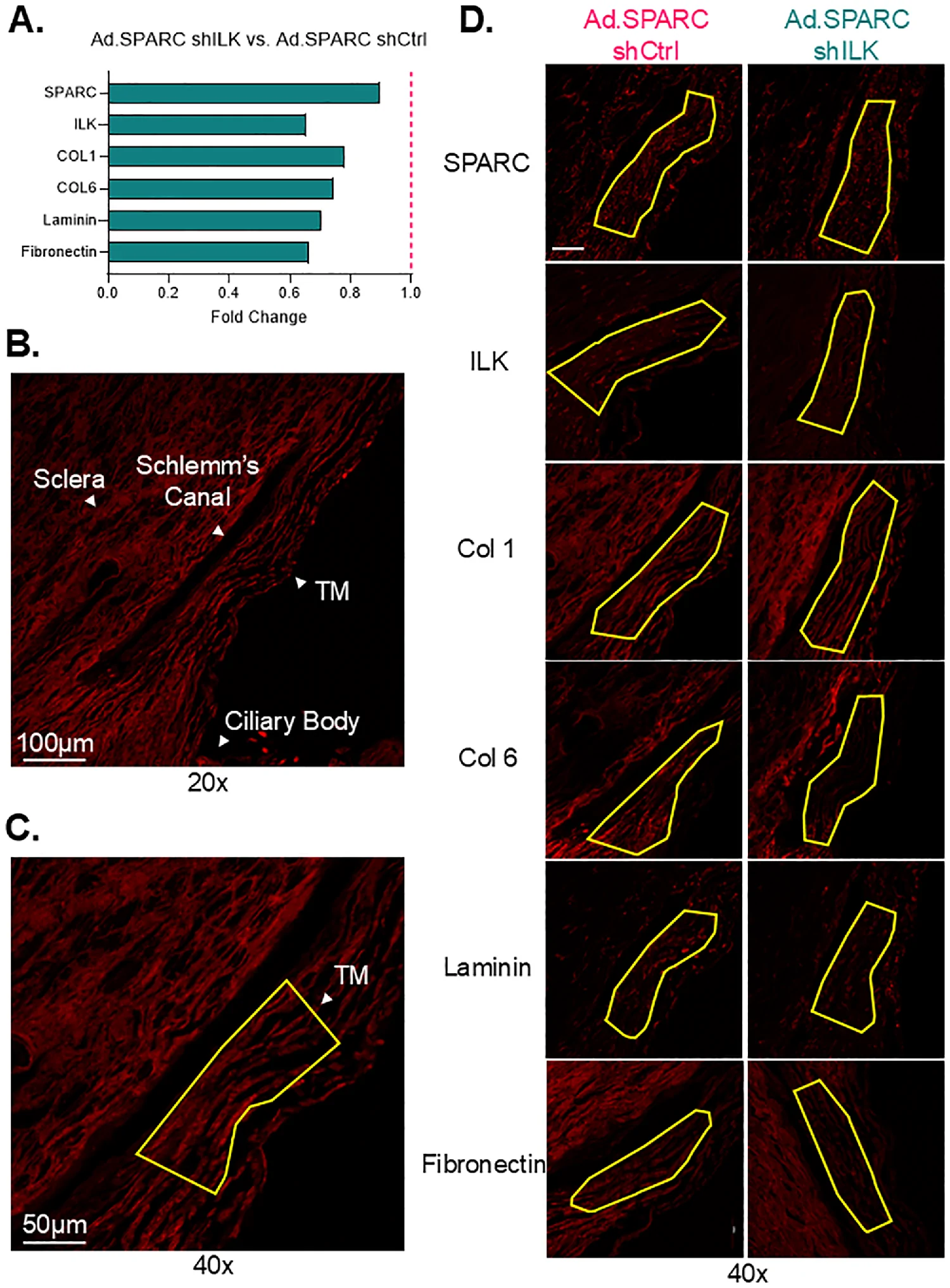
Immunostaining of ECM proteins following anterior segment injection. (**A**) Fluorescence intensity measurements were compared relative to ad.SPARC + shCtrl. (**B**) The 20× objective lens image with labeled structures. (**C**) The 2× digital zoom applied, with the *yellow bar* highlighting the TM region of interest. (**D**) Representative images with the TM highlighted. Supplementary Figure S2 shows DAPI and negative control images.

In human cadaveric anterior chambers, the injection of Ad.SPARC elevated IOP by 2.10 ± 0.25-fold (*P* = 0.021, *n* = 4). The addition of shILK blunted the effect of SPARC overexpression on IOP by 0.61 ± 0.31-fold (*P* = 0.015, n = 4) by the end of the experiment (Fig. 3). Average outflow facility decreased from 0.188 to 0.089-µL/min/mm Hg after treatment with Ad.SPARC shCtrl eyes and decreased from 0.190 to 0.128 µL/min/mm Hg after treatment with Ad.SPARC shILK. Unlike in mice, the addition of ILK suppression reduced the IOP at many time points but did not eliminate SPARC's effect. The cadaveric eyes were then immunostained showing trends in reducing ILK, Col I, Col VI, laminin, and fibronectin when compared with the eyes with only SPARC overexpressed (Fig. 4A). However, these changes were not significant, likely due to low statistical power from smaller sample sizes. Representative images identifying the TM and surrounding tissue were also labeled (Figs. 4B–D), with negative controls in Supplementary Figure S2, and co-staining of SPARC and ILK in Supplementary Figure S4.

### Effect of SPARC Overexpression and ILK Inhibition on ECM Proteins in Human TM Cells

**Figure 5.**
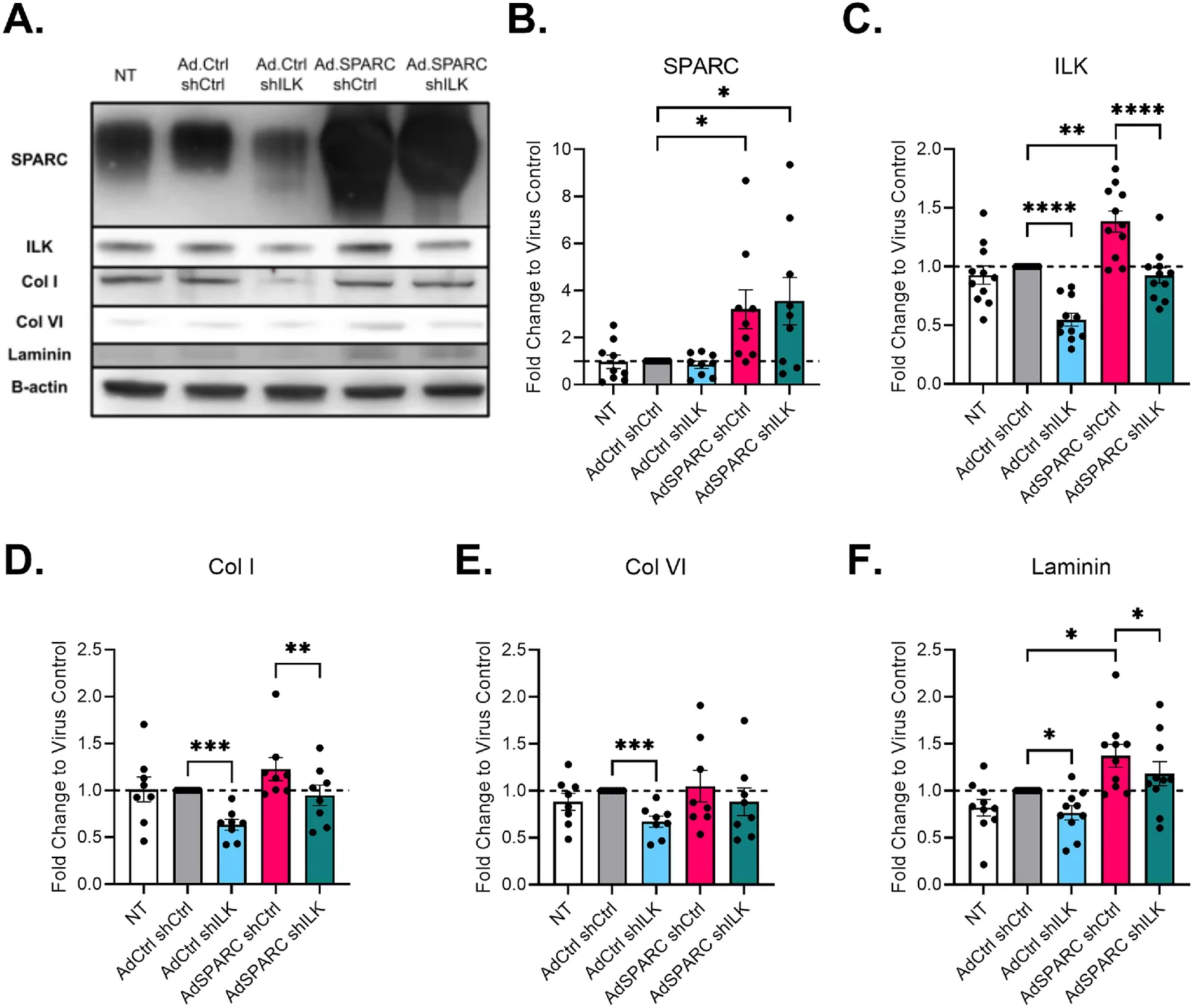
Immunoblotting of primary human TM cells. (**A**) Representative images demonstrating changes of ECM proteins after treatment with Ad.Ctrl + shCtrl (vehicle control), Ad.Ctrl + shILK (ILK suppression), Ad.SPARC + shCtrl (SPARC overexpression), and Ad.SPARC + shILK (SPARC overexpression + ILK suppression). (**B–F**) Quantification of specific ECM protein targets, fold changes were calculated compared to the vehicle control.

In human TM cells, Ad.SPARC increased SPARC expression 3.2 ± 2.5-fold (*P* = 0.029, *n* = 9) and increased ILK expression 1.38 ± 0.09-fold (*P* = 0.001, *n* = 11) compared to vehicle control, and is consistent with our lab's previous experience (Figs. 5A, 5B).^15^ Immunoblot analysis also confirmed shRNA suppression of ILK by 0.55 ± 0.05-fold (*P* < 0.001, *n* = 11). When SPARC was overexpressed alongside ILK suppression, ILK levels were only modestly reduced (0.92 ± 0.06-fold, *P* < 0.001, *n* = 11) and did not reach the lower levels achieved by ILK suppression alone (Fig. 5C). ILK inhibition alone had the greatest effect on reducing ECM protein quantity lowering levels of Col I 0.63 ± 0.06-fold (*P* < 0.001, *n* = 8), Col VI 0.67 ± 0.06-fold (*P* < 0.001, *n* = 8), and laminin 0.76 ± 0.08-fold (*P* = 0.01, *n* = 10; Figs. 5D–F). ILK inhibition also blunted SPARC overexpression by further reducing Col I 0.28 ± 0.11-fold (*P* = 0.003, *n* = 8) and laminin 0.20 ± 0.13-fold (*P* = 0.05, *n* = 10). Of consideration, ILK suppression also decreased levels of SPARC 0.85 ± 0.16 (*P* = 0.37, *n* = 9), but this change was not statistically significant. Consistently the same trend was observed, ILK inhibition showed the least protein expression, SPARC overexpression showed the greatest protein expression, and combined treatment resulted in an intermediate protein expression level. ILK inhibition alone in TM cells had a significant effect on reducing ECM protein expression, and greater effects than SPARC overexpression.

### Effect of SPARC Overexpression and ILK Inhibition on the Actin Cytoskeleton

**Figure 6.**
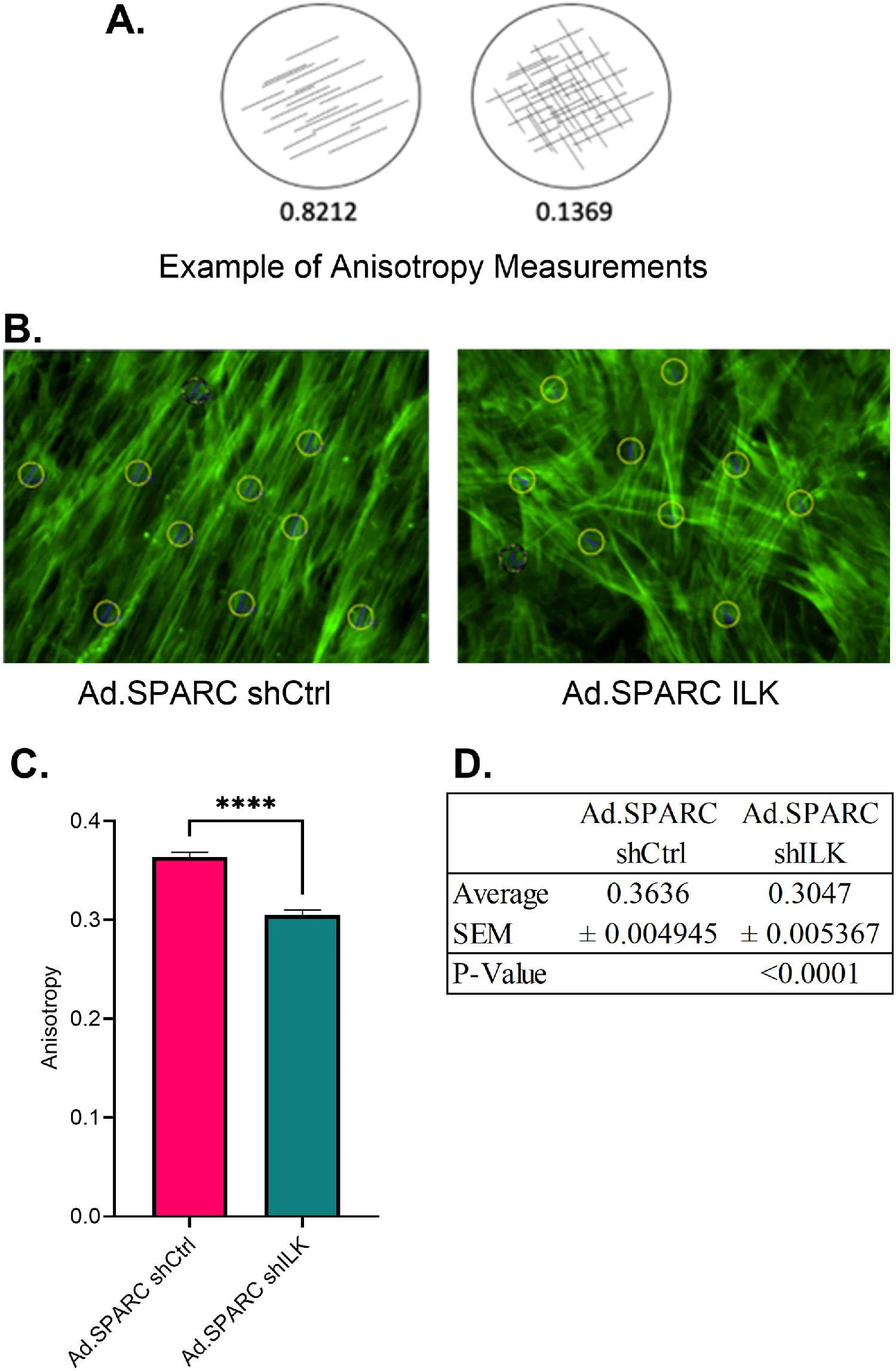
Anisotropy analysis of the structural change of f-actin in human TM cells. (**A**) Example of anisotropy measurement and calculation. (**B**) Representative images of the TM cells during the measurement process. (**C,** **D**) Quantification of the differences (*P* < 0.0001) in anisotropy measurement between cells treated with Ad.SPARC + shControl and Ad.SPARC + shILK.

The additional treatment of TM cells with shILK induced a phenotypic change in the actin cytoskeleton of cultured human TM cells. Control cultures containing Ad.SPARC and shCtrl demonstrated a linear, parallel organization of actin stress fibers, but cells treated with Ad.SPARC and shILK altered the organization of the fibers to produce a non-parallel organization throughout. The cultures treated with Ad.SPARC and shCtrl had a significantly (*P* < 0.0001) higher anisotropic value (0.3663 ± 0.0049, *n* = 8) compared with Ad.SPARC and shILK (0.3047 ± 0.0054, *n* = 8; Fig. 6). Inhibiting ILK caused a more random pattern of f-actin.

## Discussion

Our purpose was to determine the role of ILK in mediating SPARC's effect on the TM. We found that SPARC increased levels of ILK. The ILK suppression attenuated the effects of SPARC overexpression on IOP, ECM protein production, and the actin cytoskeleton implicating ILK may be a downstream mediator of SPARC's effects. SPARC is a critical regulator of the TGFβ-2 induced ocular hypertension and ILK may be the next molecular step potentially leading to a promising disease interrupting therapeutic approach.

Our data suggest ILK mediated cytoskeletal changes in TM cells as an additional mechanism, beyond ECM modulation, by which SPARC regulates outflow resistance. ECM protein accumulation disrupts the biomechanical and homeostatic mechanisms governing IOP, reducing aqueous outflow and driving IOP elevation.^34^ In combination, aging reduces α5β1 integrin expression, prompting a compensatory upregulation of αvβ3 integrin that promotes the profibrotic ECM remodeling characteristic of POAG.^35^ Notably, αv integrins regulate cell proliferation through activation of ILK, which binds to the cytoplasmic tails of β1 and β3.^36^^,^^37^ ILK knockdown impairs the ability of β3-containing integrins to maintain their active state, suggesting that ILK suppression may alter actin remodeling and ECM signaling through this integrin-dependent mechanism.^38^ SPARC has been shown to directly bind and activate ILK through the ILK-PINCH-parvin complex, linking matricellular proteins to the cytoskeleton and remodeling the ECM.^17^ ILK is crucial in managing cell-ECM adhesion structures and regulating the actin cytoskeleton.^36^^,^^39^ In mutant ILK, ECM linkage is weakened with reduced f-actin bundling.^40^^,^^41^ Reduced mature ECM development and cytoskeleton alterations enhance conventional outflow to reduce IOP and share similar capabilities to Rho kinase inhibitors currently used in glaucoma management. ILK diphosphorylates myosin light chain in smooth muscle, and thus, the inhibition of ILK may decrease cell contractility to promote a more relaxed cytoskeletal state conducive to increased outflow.^42^ The Rho kinase-specific inhibitor, Y-27632, changes cell shape, actin stress fibers, and focal adhesions, which are associated with increased flow across the JCT.^43^ Although Rao et al. did not quantify anisotropy, the appearance of the actin cytoskeleton in Y-27632 treated cells were similar to cells after ILK inhibition, suggesting enhanced paracellular flow as a mechanism of IOP reduction.^44^ Concurrent with reduced ECM deposition, the SPARC-ILK axis may regulate IOP through a dual mechanism, modulating both the intracellular cytoskeletal architecture and the ECM of the TM.^45^ A limitation in this analysis is the absence of matched NT and vehicle control groups, as the original cell lines were no longer available for experimental replication. Additionally, the effect of ILK on the cytoskeleton cannot be fully ascertained as our lab's earlier experiments overexpressing SPARC did not examine actin stress fiber organization which may be an unrecognized mechanism to SPARC's activity.

Limitations of this study include small sample sizes for cadaveric human anterior segments, reducing statistical power and limiting definitive conclusions. The scarcity and cost of donor eyes, combined with inherent inter-donor variability in ocular history and baseline outflow facility are fundamental constraints of this model. Additionally, spatial targeting to confirm viral transduction within the outflow region was not performed, limiting certainty of viral effects with the TM specifically. A future time-course analysis may help elucidate whether cross-regulatory interactions may be occurring that alter the rates of overexpression and suppression. The variability of ILK suppression on SPARC expression suggests the relationship may not be strictly unidirectional. Compensatory feedback mechanisms may sustain or variably modulate SPARC expression, and future research is needed to determine this role. Immunostaining of tissues is also subject to limitations, including a lack of standardization and potential selection bias. However, there were consistently lower expressions of ECM proteins in sections from tissues with ILK suppressed. Moreover, ILK suppression in immunoblotting appeared to have the most consistent effect on ECM proteins correlated to outflow resistance. Collagen I accumulation in the JCT region has been correlated with elevated IOP and was reduced by ILK inhibition in our study.^46^ Whole genome sequencing also identified collagen VI and laminin as key IOP elevating proteins in patients with POAG, with altered collagen VI distribution seen in glaucomatous cells and tissue.^47^^,^^48^ In addition, our results showed changes in animal models—shILK decreased collagen VI in human TM cells and anterior segments but not directly in mice. Notably, our study did not measure levels of fibronectin in human TM cells due to technical difficulties with antibody compatibility. Future studies should standardize secondary antibodies and confirm the protein-level of the reported immunostaining findings. Another potential confounding variable is that the changes observed in mice and human anterior segments could reflect alterations in TM endothelial cellularity. However, it is unlikely that minor changes in TM cellularity would result in the observed reduction in IOP. In general, our results showed that certain ECM protein expression levels mirror the relative expression of ILK and may represent how ILK suppression reduces IOP.

The effects of ILK suppression were also similar to SPARC suppression, which reduced levels of collagen I and VI in vivo and ex vivo.^14^ Additionally, ILK suppression attenuated elevated laminin levels induced by SPARC overexpression, which was not previously seen form SPARC suppression.^14^^,^^15^ In other models, ILK is responsible for laminin mediated cell-matrix interactions, suggesting a unique property of ILK on attenuating SPARC overexpression in the TM.^49^ In a future study, we plan on suppressing and overexpressing ILK alone to determine its role in TM. This may be important as ILK has been shown to paradoxically upregulate MMP-2 and MMP-9 transcription through AP-1 transcription factor dependent pathways.^50^^,^^51^ TGF-β also affects MMPs; the effect of ILK on MMPs may be context dependent and will need to be further evaluated in a future study.^52^^,^^53^ The deletion of ILK is lethal in mice and multiple animal model systems, making conditional suppression models like the Cre-Lox system promising.^54^ Additional future studies may include identifying the specific cell-types responsible for pressure attenuation by utilizing a conditional knockdown or single-cell transcriptomic profiling of TM and outflow tissues.

This study was designed as a proof-of-concept that ILK suppression modulates SPARC-driven ECM changes in the trabecular meshwork. Further examination of the underlying signaling cascade is warranted to elucidate the pathophysiology of POAG. Because ILK is partially required for the action of SPARC, we have identified it as a previously unrecognized downstream molecule in the TGF-ß-SPARC signaling pathway in the TM. We hypothesize that the receptor-based interactions with SPARC are responsible for its effects on the ECM. Understanding the molecular pathway responsible for POAG is critical for discovering new targeted therapies.

## Supplementary Material

Supplement 1
