# Supplementary material for: Integrin-Linked Kinase Suppression Reduces SPARC's Effect on Intraocular Pressure and Extracellular Matrix
Source: Invest Ophthalmol Vis Sci. 2026 Aug 21;67(10):53. doi: 10.1167/iovs.67.10.53 (PMC13505794; doi:10.1167/iovs.67.10.53)
Supplement: Supplementary file 1 [file iovs-67-10-53_s001.docx]

**Supplementary Material**

**Supplementary Figure S1.** (A-F) Expanded immunostaining of mice including negative controls for Figure 2.

**Supplementary Figure S2.** (A-F) Expanded immunostaining of human cadaveric anterior segments and their negative controls for Figure 4.

**Supplementary Figure S3.** Co-staining of murine eyes for SPARC and ILK.

**Supplementary Figure S4.** Co-staining of human cadaveric anterior segments for SPARC and ILK.
